# Autonomic nervous system and mediating role of respiratory function in patients with ALS

**DOI:** 10.1038/s41598-025-94844-y

**Published:** 2025-03-27

**Authors:** Nieves de Bernardo, José Enrique de la Rubia Ortí, Carlos Villarón-Casales, Jesús Privado, Rosa Maset-Roig, Montse Cañabate, David Sancho-Cantus, Inmaculada Orrit Sanz, Raquel Fernández Fernández, Belén Proaño, Asta Tvarijonaviciute, Camila Peres Rubio, María Benlloch, Rubén Menargues-Ramírez, Jorge Alarcón-Jiménez

**Affiliations:** 1 Department of Physiotherapy, Catholic University San Vicente Mártir, 46001 Valencia, Spain; 2 Department of Basic Biomedical Sciences, Catholic University of Valencia, 46001 Valencia, Spain; 3 Biomechanics and Physiotherapy in Sports (BIOCAPS), Faculty of Health Sciences, European University of Valencia, 46001 Valencia, Spain; 4 Department of Methodology of Behavioral Sciences, Universidad Complutense de Madrid, Campus de Somosaguas, , 28223 Pozuelo de Alarcón, Madrid Spain; 5 Department of Nursing, Catholic University San Vicente Mártir, 46001 Valencia, Spain; 6 Psychiatry Service, University Hospital Dr. Peset, 46017 Valencia, Spain; 7 IMEDUCV, Catholic University San Vicente Mártir, 46100 Burjassot, Spain; 8 Functional testing unit La Fe Polytechnic and University Hospital, 46026 Valencia, Spain; 9 Interdisciplinary Laboratory of Clinical Analysis, Campus of Excellence Mare Nostrum, University of Murcia, 30100 Murcia, Spain; 10 Nursing Department, Faculty of Health Sciences, University of Alicante, 03690 San Vicente del Raspeig, Alicante Spain

**Keywords:** Amyotrophic lateral sclerosis, Autonomic nervous system, Respiratory capacity, Functionality, Cognition, Oxidative stress, Health care, Medical research, Neurology, Signs and symptoms

## Abstract

Patients with Amyotrophic Lateral Sclerosis (ALS) exhibit altered patterns of respiratory rate and heart rhythm that are directly related to autonomic nervous system (ANS) activity. This study aimed to analyze the role of the ANS in respiratory function, cognition, functionality, and antioxidant capacity in patients with ALS through a predictive model that assesses the mediating activity of respiration. This quantitative, observational, analytical, and cross-sectional clinical study was conducted using a sample of 75 patients diagnosed with ALS. ANS activity, respiratory function, cognition, functionality, and antioxidant capacity were also measured. Using these values, a structural equation model was developed using AMOS V.23 software. The mediational predictive model showed that increased sympathetic nervous system (SNS) activity, in turn, increased respiratory function, whereas the role of the parasympathetic nervous system in respiration was very weak and had the opposite effect. Furthermore, SNS activity increased respiratory function values, which, in turn, improved functional capacity, cognition, and antioxidant power in patients with ALS, with respiratory function playing a mediating role. The mediating effect of respiratory function was observed primarily between ANS and functional disability. For oxidative stress, respiratory function showed a high mediating effect, such that greater respiratory function corresponded to greater antioxidant capacity. Additionally, for cognitive activity, a moderate direct effect of the ANS was observed; however, it was greatly enhanced by respiratory disability. Finally, differences were only found based on sex, with respiratory capacity and antioxidant power being higher in men.

## Introduction

Amyotrophic Lateral Sclerosis (ALS) is a progressive neurodegenerative disease that affects motor neurons, typically resulting in patient mortality due to respiratory failure within 3–5 years of symptom onset^1^. It is classified as a rare disease, with an annual incidence of 1.4 cases per 100,000 inhabitants, exhibiting a higher prevalence in males^2^. Clinically, it can be classified as a bulbar-onset or spinal-onset disease^3^, with the age range of 45 to 75 years being the most at risk for developing ALS^4^.

The origin of the pathology is attributed to different mechanisms, such as misfolded protein aggregation and dysfunction of protein degradation pathways, such as the ubiquitin-proteasome system and autophagy or glial dysfunction^1^, among others, and increased oxidative stress associated with mitochondrial damage^5^. Oxidative stress stems from the extensive metabolism of motor neurons and their vulnerability to damage from reactive oxygen species (ROS)^6^, currently representing one of the principal therapeutic targets for the disease^7^.

Clinically, patients with ALS exhibit progressive functional disability characterized by the loss of physical function in the bulbar, motor (especially in the arms and legs), and respiratory domains^8^. While ALS is predominantly known for its motor symptoms, autonomic nervous system (ANS) disorders have also been described, including decreased heart rate variability (HRV)^9–12^ and chronic cardiac sympathetic hyperactivity^13,14^. These alterations have been confirmed in our laboratory using Polar H7 Bluetooth technology and the ELITE HRV application^15^. The ANS, divided into the Sympathetic Nervous System (SNS) and the Parasympathetic Nervous System (PNS), regulates vital functions including, but not limited to, heart rate, digestion, and crucially, respiratory capacity. It plays a pivotal, albeit often underestimated, role in ALS progression^16,17^. Cardiac activity and vasoconstriction or vasodilation are predominantly governed by the ANS; consequently, heart rate variability (HRV) is one of the most valuable noninvasive methods for assessing ANS activity^18^. Furthermore, ANS contributes to sensory system impairment. Sensory neuropathy in patients with ALS is manifested as reduced sensory nerve conduction velocity and impaired sensory function. This sensory involvement further complicates the clinical picture of ALS and underscores the widespread effects of the disease beyond the motor system^19^.

Despite advances in understanding the motor pathology of ALS, knowledge of how the ANS contributes to respiratory complications remains limited. Current evidence suggests a complex relationship between neuronal degeneration and autonomic dysfunction^12,16,17,20^. However, the exact mechanisms through which ANS influences respiratory capacity in ALS are not yet fully understood. Moreover, ANS alterations could be related to other types of symptomatology; approximately 50% of patients also exhibit extramotor manifestations, among which frontotemporal dementia (FTD) stands out^21^, clinically characterized by behavioral changes and impairment of executive functioning and/or language deterioration^22,23^. In this regard, an association has been found between cognitive impairment and respiratory insufficiency in ALS^24^. Furthermore, it is worth noting that in other neurodegenerative pathologies or diseases with neurodegenerative components, such as multiple sclerosis or systemic sclerosis that present respiratory insufficiency, it has already been observed that respiratory deterioration is associated with low functional disability^25,26^; Therefore, for patients as well, the loss of respiratory function could explain the lack of functionality. Interestingly, it has also been observed that respiratory insufficiency and hypoxia are produced, associated with the induction of oxidative stress^27^ mainly produced by the mitochondria, through the release of the superoxide anion (O2-)^28^. This implies a relationship between respiratory capacity and the activity of antioxidant enzymes, whose link to ANS is well known^29,30^. All these aspects lead to the hypothesis that cognitive, oxidative state, and functional alterations may be linked to those of the ANS through respiratory function.

The objective of the present study was to analyze the role of ANS in respiratory function, cognition, functionality, and antioxidant capacity in patients with ALS. To this end, a novel predictive model, that studies the possible mediating role of respiratory function between the ANS and cognition, oxidative state, and functionality in these patients, is proposed. This study aimed to determine whether the ANS directly influences cognition, functionality, and oxidative state, or if it does so through respiratory function, and therefore, if the latter would mediate the effect of the ANS on other measures (cognition, functionality, and oxidative state). Additionally, based on the results of this model, we will attempt to study whether there are differences in the components of the model based on sociodemographic variables: age, sex, and type of ALS.

## Materials and methods

### Participants

75 ALS patients were evaluated, of whom 18.7% had bulbar ALS and the rest had spinal-type ALS. The age was 56.51 years (SD = 10.38 years), with a range of 28–77 years, and 60% were male. The time of ALS diagnosis in months was 28.28 (SD = 27.69 months), with a range between 2 and 146 months. The ALSFRS-R score was 28.37 (SD = 8.68), with a range between 6 and 46.

### Procedure

A selective and cross-sectional design was employed in which participants were selected based on the characteristics relevant to the study.

To obtain the sample, the main ALS associations in Spain were contacted and informed of the project through their coordinators, who communicated it to the patients at each center. Subsequently, those who voluntarily agreed to participate were included in the study. Specifically, the inclusion criteria were as follows: males over 18 years of age, non-fertile females over 50, or between 18 and 50 years of age that were not planning to become pregnant; patients diagnosed and symptomatic with ALS for at least 6 months prior to inclusion in the study; patients treated with Riluzole; Acceptance of participation in the study by signing the informed consent form. The exclusion criteria were as follows: tracheostomy; invasive or non-invasive ventilation with positive ventilatory pressure; participation in any other trial or having done so in the 4 weeks prior to inclusion; patients with evidence of dementia; patients with alcohol or drug abuse dependence; patients infected with hepatitis B or C, positive human immunodeficiency virus; renal patients with creatinine 2 times higher than normal markers 30 days before inclusion; and hepatic patients with liver markers (ALT and AST) elevated 3 times above normal levels 30 days before inclusion. After applying these criteria, the final sample consisted of 75 patients diagnosed with bulbar or spinal ALS.

### Instruments

A series of tests and scales was applied to this sample to determine the following variables:

### ANS

To assess ANS activity, the Polar H7 Bluetooth device, an instrument that measures heart rate and can be connected to Bluetooth devices compatible with heart rate measurement software, was used. A specific application is required to view the heart rate data of the receiving device. To measure HRV, an independent application called Elite HRV connected to a Polar H7 chest strap was used. Specifically, a series of variables were determined, one of which was related to SNS. It is low heart frequency (LF), in which sympathetic mechanisms predominate^31^. Those related to the PNS calculated were heart rate variability index (HRV), which represents the variation over time between RR intervals in the electrocardiogram, defined as the physiological variation in the duration of the interval between each heartbeat^32^; the root mean square of the sum of squares of differences between adjacent RR intervals (RMSSD)^31,32^; the natural logarithm of the RMSSD to distribute the figures in a more easily understandable interval (RMSSD LN)^33^; the percentage of consecutive RR intervals that differ from each other by more than 50 ms (PNN50)^34^; and high heart frequency (HF power)^35^.

### Respiratory function

There are different procedures for the measurement of lung function in patients with ALS, among which spirometry stands out^36^. Three pulmonary function parameters were evaluated (expressed as the percentage of predicted values (%), except for forced expiratory volume in 1 s (FEV1)/FVC presented as the measured value): forced vital capacity (FVC), FEV1, and FEV1/FVC^37^. For their determination, a MasterScreen PFT spirometer powered by SentrySuite™ (Jaeger, Germany) was used, and the spirometer was calibrated before measurement following established protocols for this purpose. Three acceptable and repeatable trials were performed, and the best trial was chosen^38^.

### Cognition

The Edinburgh Cognitive and Behavioral ALS Screen (ECAS) (Spanish version) was used to differentiate between various common profiles associated with aging, such as depression, Alzheimer’s disease, and frontotemporal dementia. It specifically evaluates executive function, memory, language, visuospatial ability, and social knowledge. The maximum total score is 136. It has high convergent validity with other ALS screening tests and adequate internal validity scores^39,40^.

### Antioxidant capacity

Fasting blood samples were collected to determine the antioxidant capacity of the analytes. The samples were centrifuged at 1500 g for 5 min, and the serum was separated and frozen at -80 °C until measurement. The analytes related to antioxidant capacity measured were Trolox equivalent antioxidant capacity (TEAC), Cupric ion reducing antioxidant capacity (CUPRAC) and Ferric reducing ability (FRAP). These analytes were measured using an automated biochemical analyzer (Olympus AU600, Olympus Europe GmbH, Germany), as described by Rubio et al.^41^.

### Functionality

To evaluate functionality, the revised ALS Functional Rating Scale (ALSFRS-R) was used. It is a sensitive, accurate, and reproducible scale that assesses functional capacity considering the domains of deterioration: bulbar, upper limb, lower limb, and respiratory^42^.

### Data analysis

First, descriptive statistics of the different measures employed were calculated using the SPSS V. 23 statistical package. Two confirmatory models were estimated using AMOS V. 23^43^ to study the mediating role of respiratory function between the ANS and the other measures (cognition, functionality, and oxidative state). Three types of goodness-of-fit indices were considered for the estimated models: (1) absolute, which evaluates the fit of the proposed theoretical model to the empirical data. These included the χ^2^/df index^44^, whose values less than 3 indicate a good fit to the data; the Goodness-of-Fit Index (GFI)^45^, with values > 0.95 considered a good fit; and the Standardized Root Mean Square (SRMR)^46^ and the Root Mean Squared Errors (RMSEA)^47^, with values < 0.08, indicating a good fit^48^. Additionally, the presence of < 5% standardized residuals exceeding 2.58 in absolute value is considered a criterion for a good fit^45,48^. (2) Incrementality was used to compare the obtained model with a null model. These indices include the Normed Fit Index (NFI)^44^, Comparative Fit Index (CFI)^49^, and Tucker-Lewis Index (TLI)^50^, with values > 0.95, indicating a good fit. And (3) Parsimony, evaluates the fit of the model against the number of estimated parameters, penalizing the use of more parameters. The Parsimony Goodness-of-Fit Index (PGFI)^45^, Parsimony Normed Fit Index (PNFI)^51^, and Parsimony Comparative Fit Index (PCFI)^49^ were used, with values > 0.50 indicate a good fit. A recommendation of 10 participants per indicator has been suggested^52^. However, others have proposed using only five participants per indicator when the distribution is normal^48^. In our study, there were 75 participants for the six indicators in the tested models (75/6 = 12.5). Third, to study the role of sociodemographic variables in the measures that constitute the model, Pearson’s correlation was calculated for age using these measures, and independent sample t-tests were conducted to assess whether there were differences based on age, sex, and ALS type for these measures. These analyses were performed using the SPSS version 23 statistical package.

### Ethical considerations

The project was approved by the Clinical Research Ethics Committee of Hospital de la Fe in Valencia, Spain (2021 − 001989 38), in compliance with the Declaration of Helsinki. All patients provided informed consent before inclusion in the study.

## Results

### Descriptives

Table 1 shows the descriptive statistics of the different measures used and the factors to which they belong when estimating the confirmatory models.

**Table 1:** Values obtained after measuring the variables: autonomic nervous system (ANS) activity, respiratory function, cognitive status, functional capacity, and antioxidant capacity.

Factor	Variables	Mean	SD
Sympathetic ANS	Frecuency LF Peak HZ	0.08	0.05
Parasympathetic ANS	HRV	42.69	11.56
	Frecuency Total power ms2	850.06	2269.45
	HRV RMSSD ms	22.38	21.16
	HRV LN RMSSD ms	2.77	0.75
	HRV PNN50	4.93	10.06
	Frecuency HF Power ms2	377.52	1190.68
Respiratory function	FVC	2.31	1.08
	FEV1	1.76	0.81
	FEV1%M	75.23	11.57
Functional capacity	ALSFRS-R	28.37	8.68
Cognitive state	ECAS Lenguage	25.64	3.12
	ECAS Executive	36.17	7.18
	ECAS Memory	16.54	4.25
	ECAS Visiospatial	11.57	1.28
Antioxidant capacity	TEAC mmol Trolox equivalent/L	1.05	0.13
	CUPRAC mmol Trolox equivalent/L	0.73	0.07
	FRAP mmol Trolox equivalent/L	2.45	0.39

ALSFRS-R: Revised Amyotrophic Lateral Sclerosis Functional Rating Scale; CUPRAC: Cupric ion Reducing Antioxidant Capacity; ECAS: Edinburgh ALS Cognitive and Behavioral test; FEV1: Forced expiratory volume in 1 s; FRAP: Ferric reducing antioxidant power; FVC: Forced Vital Capacity; HF: High Heart Rate; HRV: Heart Rate Variability; HRV RMSSD: The Root Mean Square of the Sum of Squares of the Differences Between adjacent RR intervals in the cardiac variability; PNN50: Percentage of consecutive RR intervals that differ from each other by more than 50 ms in cardiac variability; TEAC: Trolox equivalent antioxidant capacity.

### Confirmatory models

We followed Holmbeck^53^ and Ato and Vallejo^54^ to test the mediation model that analyzed the mediating role of respiratory function between the ANS and other measures obtained in patients with ALS. One model included the mediational effects (indirect) of respiratory function between the ANS and cognition, functionality, and oxidation (restricted model), and the other included both direct and indirect effects between the ANS and other measures (unrestricted model). In both cases, the models were estimated using the maximum likelihood due to multivariate normality, as the Bollen-Stine bootstrap procedure^55^ was not statistically significant (*p* = 0.214 for the unrestricted model and *p* = 0.164 for the restricted model).

**Fig. 1:**
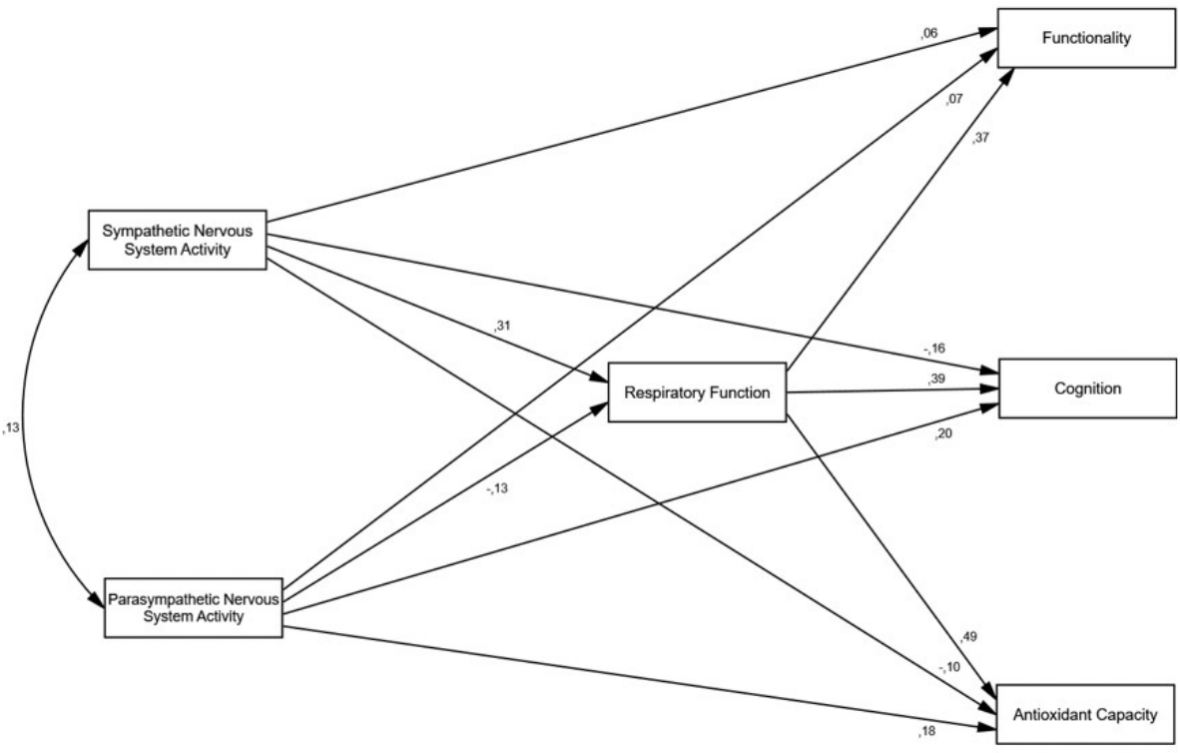
Unrestricted model. Model of direct and indirect effects of the sympathetic nervous system (SNS) and parasympathetic nervous system (PNS) on functionality, cognition, and antioxidant capacity through respiratory function.

**Fig. 2:**
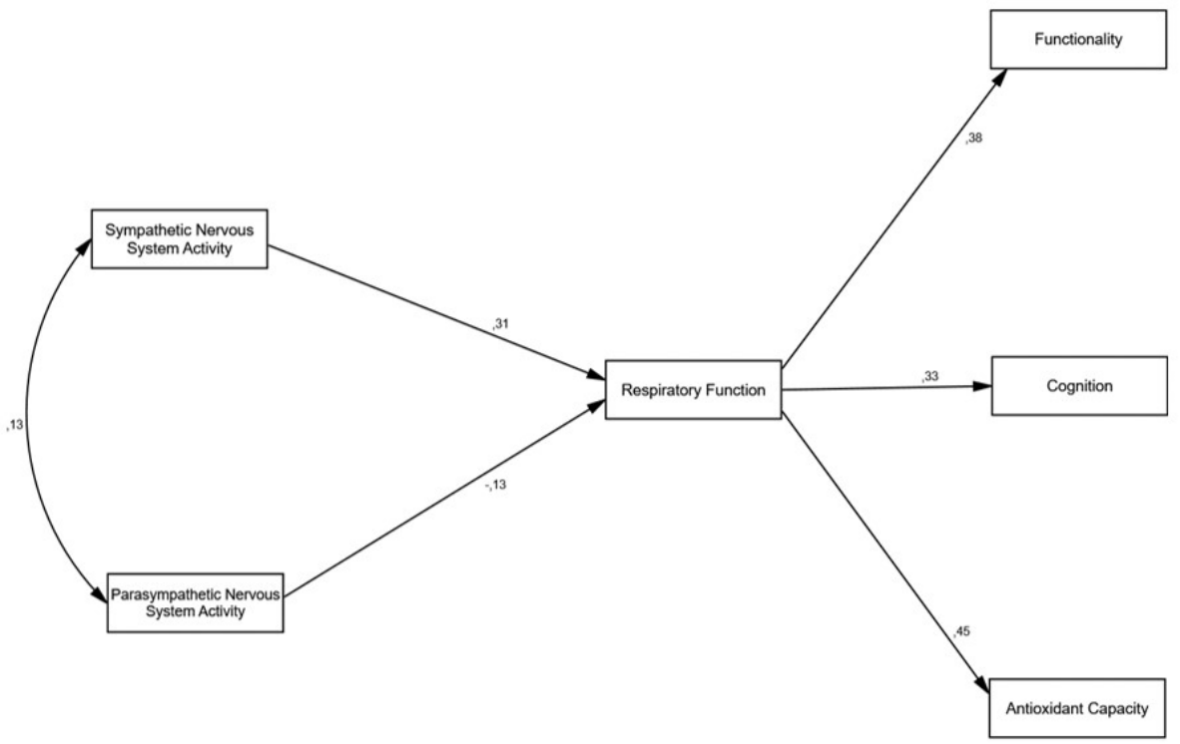
Mediational or restricted model. Model of indirect effects of the sympathetic nervous system (SNS) and parasympathetic nervous system (PNS) on functionality, cognition, and antioxidant capacity through respiratory function.

Figures 1 and 2 show the unrestricted and restricted models, respectively. Table 2 presents the fit indices for both models. The fit of both models to the data was adequate for most of the indices. When comparing both models, no statistically significant differences were found based on the number of parameters (Δχ^2^_6_ = 9.04, *p* = 0.172). Therefore, the restricted model^53,54^ should be selected, which includes indirect effects of the ANS on cognition, functionality, and oxidation through respiratory function, which indicates that it is more plausible to assume a mediating role of respiration between the ANS and other measures.

**Table 2:** Goodness-of-fit indices for the contrasted models.

	χ^2^/df	GFI	NFI	CFI	TLI	RMSEA	SRMR	Residues ≥ |± 2.58|	PGFI	PNFI	PCFI
Unrestricted	1.55	0.979	0.922	0.963	0.815	0.086	0.046	0.00%	0.140	0.184	0.193
Restricted	1.52	0.943	0.769	0.895	0.824	0.084	0.080	0.00%	0.404	0.462	0.537

GFI: Goodness of Fit Index; NFI: Normed Fit Index; PCFI: Parsimony Comparative Fit Index; PGFI: Adjusted Parsimony goodness of fit index; PNFI: Normalized Parsimony Fit Index; RMSEA: Root Mean Squared Errors; SRMR: Standardized Root Mean Square; TLI: Tuker- Lewiss Index.

Analyzing the mediational or restricted model according to Cohen’s criterion^56^ in linear regression, a value of f^2^ = R^2^/(1-R^2^) of 0.02 indicates a small effect size, 0.15 medium, and 0.35 large. Solving for the value of r in the formula, we found that *r* = 0.14 would be a small effect size, 0.36 medium, and 0.51 large. Therefore, in this mediational model, we observed that an increase in SNS activity increased respiratory function (β = 0.31), whereas the role of the SNP in respiration was very weak and had the opposite effect, as an increase in its function represented lower respiratory capacity (β = -0.13), with both systems explaining 10% of respiratory function (R^2^ = 0.10). Respiratory function positively explained the functional capacity of patients with ALS (β = 0.38), cognition (β = 0.33), and antioxidant capacity (β = 0.45). In other words, SNS activity increases respiratory function values, and this improvement in respiratory function enhances functional capacity, cognition, and antioxidant power in ALS patients, with respiratory function playing a mediating role.

### Sociodemographic variables

Pearson correlations between age and the different factors of the confirmatory model were not statistically significant: sympathetic ANS (*r* = 0.10, *p* = 0.419), parasympathetic ANS (*r* = 0.19, *p* = 0.105), respiratory function (*r* = -0.05, *p* = 0.678), cognitive state (*r* = -0.16, *p* = 0.171), antioxidant capacity (*r* = -0.02, *p* = 0.887), and functional capacity (*r* = 0.06, *p* = 0.588). Additionally, the correlation values showed a small effect size according to Cohen’s criteria^56^: 0.10, 0.30, and 0.50. Therefore, we can conclude that age does not appear to be related to measures in the model.

The results of the sex differences according to the independent samples t-test for the different measures of the model are presented in Table 3. Statistically significant differences were found only for respiratory function (favoring males) and antioxidant capacity (favoring males). Furthermore, in both cases, the effect sizes of the differences were large according to Cohen’s criteria^56^: 0.20, 0.50, and 0.80.

On the other hand, Table 3 also shows the differences based on ALS type for the model measures. In this case, there were no statistically significant differences in either group, although there was a difference with a medium effect size (d = 0.50), specifically for antioxidant capacity, with higher values for the spinal type.

**Table 3:** Differences according to sex and type of ALS for the predictive model measures.

Factor	Sex		
	Mean (SD)	t	d
Sympathetic ANS	Male: 0.01 (0.06) Female: -0.01 (0.02)	t_72_ = 1.48, *p* = 0.143	0.35
Parasympathetic ANS	Male: 1.88 (25.59) Female: -2.96 (12.05)	t_72_ = 0.95, *p* = 0.344	0.23
Respiratory function	Male: 0.31 (0.83) Female: -0.48 (0.57)	t_72_ = 4.45, *p* < 0.001	1.06
Cognitive state	Male: 0.56 (4.31) Female: -0,95 (6.09)	t_72_ = 1.25, *p* = 0.217	0.30
Antioxidant capacity	Male: 0,03 (0.06) Female: -0.04 (0.05)	t_72_ = 4.58, *p* < 0.001	1.09
Functional capacity	Male: 29.13 (8.04) Female: 27.17 (9.76)	t_72_ = 0.94, *p* = 0.350	0.22

Factor	Type of ALS		
Sympathetic ANS	Spinal: -0.00 (0.06) Bulbar: 0.00 (0.03)	t_72_ = -0.15, *p* = 0.882	-0.04
Parasympathetic ANS	Spinal: -1.37 (16.75) Bulbar: 5.79 (35.23)	t_72_ = -1.13, *p* = 0.261	-0.36
Respiratory function	Spinal: 0.04 (0.78) Bulbar: -0.18 (1.01)	t_72_ = 0.87, *p* = 0.366	0.26
Cognitive state	Spinal: -0.15 (5.13) Bulbar: 0.45 (5.09)	t_72_ = -0.39, *p* = 0.696	-0.12
Antioxidant capacity	Spinal: 0.01 (0.06) Bulbar: -0.03 (0.08)	t_72_ = 1.69, *p* = 0.096	0.50
Functional capacity	Spinal: 28.98 (8.35) Bulbar: 25.71 (10.16)	t_72_ = 1.27, *p* = 0.210	0.38

## Discussion

Autonomic dysfunction, also known as dysautonomia, is a facet of ALS that deserves further exploration because of its direct impact on patients’ quality of life and survival^17^. Limited published research has documented variations in the function of the autonomic nervous system (ANS) in patients with ALS, observing alterations in HRV and cardiovascular response, suggesting an imbalance between sympathetic and parasympathetic activity^12,16,17^. This imbalance, as previously observed in our laboratory for the same population, is characterized by sympathetic nervous system hyperactivity relative to that of the parasympathetic nervous system^15^, as previously described by other authors^13^. Additionally, the progressive nature of autonomic dysfunction in ALS has been observed using various measures such as heart rate variability and questionnaires assessing autonomic symptoms. Specifically, Dubbioso et al. (2023) found that autonomic dysfunction, assessed using composite autonomic symptom scores, is associated with disease progression and survival in ALS^57^. In this study, the authors also showed a correlation between autonomic dysfunction and clinical milestones, such as reaching King’s stage 4. These findings support our observations and underscore the importance of considering autonomic dysfunction in ALS management.

In the current study, we attempted to delve deeper into the consequences of dysautonomia on certain functions that are altered in ALS. As described in the results, as there were no significant differences between the unrestricted model showing the direct and indirect effects of the ANS (Fig. 1) and the restricted model showing the indirect effects of the ANS (Fig. 2), our results should be adjusted to the second model, showing a clear mediating effect of respiratory insufficiency. However, to compare the direct and indirect effects of the ANS on cognition, functionality, and oxidative status in patients with ALS, we compared the results obtained using both models. In terms of functionality, our model did not indicate a direct impact of the sympathetic nervous system (β = 0.06) or the parasympathetic nervous system (β = 0.07). However, the effect size of respiratory function on functionality was of a large and positive magnitude (β = 0.37) (Fig. 1), which was confirmed in the restricted model (Fig. 2) with a slightly larger effect size (β = 0.38). Therefore, deterioration of respiratory function appears to be particularly relevant, consistent with the findings in other diseases that involve respiratory insufficiency^58^.

Regarding cognitive impairments, there is no scientific evidence that they are due to the ANS, but rather to the significant role of respiration^24^. However, our results show a medium direct influence of sympathetic and parasympathetic activity (β = − 0.16 and β = 20, respectively) (Fig. 1), albeit with opposite signs; hence, higher sympathetic system activity leads to lower patient cognition capacity, while higher parasympathetic activity leads to better cognition. Despite this relationship, the effect was much greater when considering the effect of the ANS on respiration, both in the unrestricted model (with an effect of β = 0.39) and in the restricted model (β = 0.33).

Finally, it has been confirmed that oxidative stress in certain pathologies is promoted by increased sympathetic activity^30^. In line with this, in our unrestricted model, there was a weak direct negative relationship (β = − 0.10) between the sympathetic nervous system and the patient’s antioxidant capacity. However, this relationship became strong and positive (β = 0.49) when evaluated through respiratory function, a finding confirmed in the restricted model with a slightly lower value (β = 0.45) (Fig. 2). This could indicate that the direct effect of the sympathetic system is reversed through respiration, while the parasympathetic effect may be modulating in ALS patients (β = − 0.13) (Fig. 1), as seen in exacerbated COPD patients^59^, where worse respiratory function leads to higher parasympathetic activity.

Therefore, in our model, what appears most relevant to the disease is the relationship between autonomic dysfunction, primarily involving the sympathetic nervous system (especially hyperactive in these patients), and respiratory insufficiency (which in turn represents one of the main causes of death in ALS)^12,20^. This effect has already been observed in patients with COPD, who have a higher risk of cardiovascular disease (CVD)^60^ and show worsening VFC compared to healthy individuals^61^. This clear relationship between both variables seems to justify the hypothesis proposed in this study regarding the potential mediating role of respiratory function between ANS activity and cognition, antioxidant capacity, and functional ability.

Regarding the possible influence of sex, age, and ALS type (bulbar or spinal) on the model variables, differences were found only based on sex, with respiratory capacity and antioxidant power being higher in men.

Thus, our results support the hypothesis that autonomic dysfunction in ALS occurs in a manner analogous to the alteration of motor neurons, which is in turn a consequence of the combination of degeneration within the central structures^62,63^. y periféricas^64,65^. Furthermore, our results showed a positive relationship between sympathetic activity and respiratory function, which could be primarily attributed to the increase in plasma noradrenaline, which has been previously described in these patients^66,67^. In turn, deterioration of respiratory function has already been associated with greater functional disability^68^, which could ultimately be explained by an increase in muscle fatigue resulting from a lack of oxygen in the blood. Regarding the hypothesis that respiratory function serves as the link between ANS activity and cognitive capacity, it is worth noting that autonomic dysfunction has also been described in frontotemporal lobar degeneration^69^. This is why respiratory system disorder may precisely be the link between both variables, as it is the leading cause of death in patients with frontotemporal lobar degeneration^70^. Finally, impaired respiratory function is associated with oxidative stress^71^. In this regard, pathways that generate reactive oxygen species (ROS)^72,73^ and those that promote reactive nitrogen species (RNS) in the human lung are well described in response to injury^74^.

This finding, combined with the existing literature on the role of oxidative stress in ALS^5,7^, suggests that therapies aimed at improving respiratory function and/or enhancing antioxidant capacity may be promising for mitigating disease progression. For example, non-invasive ventilation and/or antioxidant supplementation have already been used with promising results^75^.

Regarding the limitations of the study, it is worth noting that a small population sample was used; furthermore, the means of heart rate variability showed a lot of variance, thus being unstable. In this sense, it should be taken into account that individuals who agreed to participate in clinical studies may differ systematically from the broader ALS population; that is, we assume that the inherent limitations of voluntary participation mean that some degree of selection bias cannot be entirely ruled out. Therefore, further research in larger, more diverse cohorts is needed to confirm the generalizability of our findings to a wider ALS population. In addition, it would be of interest to further delve into this analysis, adding other biomarkers of oxidative stress and inflammation, among others, to help understand the role of the ANS in ALS. In this sense, we have not taken into account the recent role of the endocannabinoid system, including the profile of circulating lipid mediators, as potential biomarkers of disease progression in ALS. Future studies should explore the potential connection between neuroinflammation, energy metabolism, and the endocannabinoid system in ALS. Several studies^76,77^, have explored the role of endocannabinoids in ALS, suggesting potential therapeutic avenues. Further research incorporating analyses of circulating lipid mediators could provide valuable insights into the complex pathophysiology of ALS, and potentially identify novel therapeutic targets. We also propose to assess the role of sex in the association between variables. Finally, another limitation of this study is the use of systemic measurements of antioxidant capacity, which reflect overall bodily status rather than specifically intraneuronal processes. Future studies could benefit from prospective studies involving long-term follow-up, de oxidant/antioxidant alterations in cerebrospinal fluid (CSF), or conducting cellular experiments using motor neurons derived from induced pluripotent stem cells (iPSCs) of ALS patients to gain a more direct understanding of neuronal oxidative stress.

Based on this study, it can be concluded that the relationship between the sympathetic nervous system and respiratory capacity is high and positive, whereas the relationship between the parasympathetic system and respiratory capacity is low and negative. Regarding functional disability, it appears that the effect of the ANS is primarily due to the mediating effect of respiration, whereas for cognitive activity, there is a moderate ANS effect that is greatly enhanced by respiratory impairment. Finally, ANS, through sympathetic activity, appears to weakly promote oxidative stress directly, although this effect is reversed when evaluating the mediating effect of respiration. In this way, higher respiratory function leads to greater antioxidant capacity and, therefore, lower oxidative stress, with the role of the parasympathetic system apparently being modulating in nature. These conclusions highlight the therapeutic importance of complementing current pharmacological treatments with therapies aimed primarily at improving respiratory function.

## Data Availability

The data that support the findings of this study are available on request from the corresponding author. The data are not publicly available due to privacy or ethical restrictions.
